# Health-related quality of life in porphyria cutanea tarda: a cross-sectional registry based study

**DOI:** 10.1186/s12955-020-01328-w

**Published:** 2020-03-30

**Authors:** Janice Andersen, Janne Thomsen, Åshild Rostad Enes, Sverre Sandberg, Aasne K. Aarsand

**Affiliations:** 1 Department of Medical Biochemistry and Pharmacology, Norwegian Porphyria Centre (NAPOS), Haukeland University Hospital, N-5021 Bergen, Norway; 2 Norwegian Organization for Quality Improvement of Laboratory Examinations, Haraldsplass Deaconess Hospital, N-5009 Bergen, Norway

**Keywords:** Porphyria cutanea tarda, Quality of life, Photodermatosis, Porphyria, SF-12, Registry, HRQoL

## Abstract

**Background:**

Porphyria cutanea tarda (PCT) is a rare, photosensitive disease characterized by skin fragility and blistering on sun-exposed areas. There is little previous research on how this condition affects health-related quality of life (HRQoL) and to the best of our knowledge this is the largest sample of PCT patients surveyed about their HRQoL. The aims of this study were to describe HRQoL, symptoms, susceptibility factors, disease activity and treatment in patients with PCT, and investigate the associations between these factors.

**Methods:**

This is a cross-sectional, retrospective study based on patient-reported outcome and laboratory data. The Norwegian Porphyria Centre diagnoses all patients with PCT in Norway, all of whom are invited to participate in the Norwegian Porphyria Registry. Between December 2013–2015, 111 patients received a postal questionnaire and invitation to participate.

**Results:**

Sixty-eight persons responded, with seven being excluded due to prolonged response time or missing information, resulting in 61 participants in the final analyses (55%). Median age was 60 years and 33 were female. We found a moderate negative relationship between the type and localisation of PCT symptoms and both mental (*r* = −.354 *p* < 0.01) and physical (*r* = −.441, *p* < 0.01) aspects of HRQoL. Participants who had started treatment when answering the questionnaire reported significantly better physical functioning and less bodily pain than those who had not started treatment. We did not observe an association between biochemical markers of disease activity and symptoms or HRQoL. Itching, a symptom that has received little attention in PCT was reported by 59% of the participants.

**Conclusions:**

Our results show that reduced HRQoL is associated with more symptoms and not having started treatment. PCT is a rare disease, and there is a need for the development of best-practice guidelines to facilitate good patient care.

## Introduction

Porphyria cutanea tarda (PCT) is the most common form of porphyria worldwide, with an estimated prevalence of symptomatic disease of 10:100000 [1, 2]. The disease is caused by an inherited (familial PCT) or acquired (sporadic PCT) defect in the haem biosynthesis enzyme uroporphyrinogen decarboxylase (UROD), which leads to excess production of uroporphyrinogen and other carboxylated porphyrinogens in the liver. Accumulation of the corresponding water-soluble porphyrins in the skin causes cutaneous photosensitivity [3].

Clinically, the condition is characterized by skin fragility, blistering, milia, hypertrichosis and hyperpigmentation on sun-exposed areas. Familial PCT is inherited in an autosomal dominant pattern with low clinical penetrance and has a higher prevalence in Norway than other countries because of founder mutations [4]. Susceptibility factors for both PCT types are iron overload, hepatic infections—in particular, hepatitis C—high alcohol consumption, HIV, smoking and the use of oestrogens [4–6]. PCT may become symptomatic at any age, but typically the age of symptom debut is middle-age [1]. Treatment usually comprises weekly or biweekly phlebotomy to reduce the iron overload or low-dose chloroquine, which mobilizes porphyrins from the liver, or a combination of the two [5, 7]. Treatment usually leads to resolution of blistering within two to four months, but complete clinical and biochemical remission may take up to 13 months [7, 8]. Prognosis is good with an estimated relapse rate of 5–17 per 100 person years [3]. Annual follow-up visits are recommended to avoid and detect relapses, and these visits should include clinical evaluation, assessment of patient compliance with control of susceptibility factors, and measurement of iron and porphyrin levels [3, 5].

Generally, optimal therapeutic outcomes and patient satisfaction require knowledge of patients’ concerns and expectations. Such subjective perspectives must be evaluated using patient reported outcome (PRO) data. For dermatological diseases, the evaluation of quality of life (QoL) is particularly important because of the visible aspect of these conditions and the potential psychological impact [9]. Our previous work in patients with PCT found that, at their worst, PCT symptoms can be very dramatic and patients may perceive PCT as a chronic and systemic disease [10, 11]. To our knowledge, the present study is the largest sample of PCT patients surveyed about health-related QoL (HRQoL), and only one previous study on QoL in people with PCT has been published. However, this included only a small number of participants (*n* = 12) who most likely were in clinical remission [12]. This highlights the need for further studies into HRQoL and the use of PRO in patients with PCT.

The aims of the present study were to describe HRQoL, symptoms, susceptibility factors, disease activity and treatment in patients with PCT, and investigate the association between these factors.

## Methods

### Design

The present study was a retrospective cross-sectional study based on PRO data from the Norwegian Porphyria Registry and laboratory data from Haukeland University Hospital.

### The Norwegian Porphyria Registry and recruitment

The Norwegian Porphyria Registry was established in 2002 and gained status as a national medical quality registry in 2012. This status is awarded by the Norwegian Directory of Health when there is a need for structural data on treatment and follow-up for the disease(s) in question, and the registry fulfils a set of formal requirements [13]. In short, these encompass that the registry has documented the legal basis for processing of personal data, that it has received data processing authority and financial support from their Regional Health Authority and that it has secured stakeholders’ commitments. Furthermore, it must have statutes detailing user involvement and data delivery and to be able to document coverage of the patient population they serve [13]. Questionnaires included in the registry have been developed in collaboration with patients, through input from patient organisations, or from issues raised in focus groups [10].

The Norwegian Porphyria Centre (NAPOS) diagnoses all patients with PCT in Norway, all of whom are invited to participate in the registry. Patients are usually informed of their diagnosis and receive the questionnaire from three to ten weeks after having donated the samples which resulted in the porphyria diagnosis. Participation in the registry includes completing a baseline questionnaire, which they receive together with the registry invitation. From December 2013 to December 2015 this questionnaire included the Short Form-12 Health Survey version 2 (SF-12). Participants who returned questionnaires from this period were included in the present study, with the following exclusion criteria; (i) > 6 months from the time of donating samples for porphyria analyses to answering the questionnaire and (ii) missing data about treatment. The median number of days from sampling to completing the questionnaire was 70 (range 21–167 days).

### Measures

#### Health-related quality of life

We used the SF-12 to assess HRQoL, which is defined as a generic measure of aspects of health considered to be important when describing individuals experiencing a specific condition. The instrument includes eight domains (physical functioning, physical role, bodily pain, general health, vitality, social functioning, emotional role and mental health), which are measured on a three- or five-point scale. Lower scores indicate a worse evaluation of the health aspect in question ([14] p. 14-15). The instrument also includes two component summary factors, physical and mental. In the *Physical Component Summary* (*PCS*), low scores indicate limitations in physical functioning, a high degree of bodily pain and poor general health. In the *Mental Component Summary* (*MCS*), a low score indicates frequent psychological distress, social disability due to emotional problems, low energy and poor general health ([14] p. 18).

#### Symptoms, susceptibility factors and treatment

Participants reported symptoms they had in connection with their PCT as categorical data in the form of a 5 × 5 table, i.e. type of symptoms; skin blisters, sore/fragile skin, abnormal hair growth, itching, and increased pigmentation, and their localization; face, arms/hands, legs/feet, upper body (including the neck) and other sites. Data on possible susceptibility factors and whether treatment had been initiated, the month and year of initiation, and type of treatment (phlebotomy and/or medication such as chloroquine) were also recorded. Smoking was reported using the following options: daily smokers, occasional smokers or never smoker. In this study, the designation “smoker” was given to daily smokers and those who reported occasional smoking. Alcohol consumption was recorded according to the units of alcohol consumed each week as 0, < 1, 1–5, 6–10, 11–15, 16–20, > 20. Low to moderate alcohol consumption was defined as 0–10 units per week and moderate to high alcohol consumption as ≥ 11 units per week.

#### Disease activity and laboratory analyses

The diagnosis of PCT was established by standard diagnostic algorithms based on an analysis of urinary, plasma and faecal porphyrins as previously described [4]. Briefly, the diagnosis of PCT is based on demonstration of increased concentrations of urinary uro- and heptaporphyrins, after exclusion of hereditary coproporphyria and variegate porphyria by analysis of faecal coproporphyrin III:I ratio (normal in PCT) and plasma fluorescence scanning (maximum emission wavelength < 623 nm in PCT), respectively [15]. The analyses were performed at the Department of Medical Biochemistry and Pharmacology, Haukeland University Hospital. As an indicator of disease activity at the time of diagnosis, urinary uroporphyrin was analysed by high-performance liquid chromatography, expressed per mmol creatinine [4]. PCT was classified as familial or sporadic based on sequencing of the *UROD* gene, which was performed at the Department of Medical Genetics, Haukeland University Hospital.

### Scoring and statistical analyses

We scored the SF-12 using both norm-based scoring (NBS) and, after transformation, a score in the range of 0–100 [14]. After linear transformations of scores, NBS appears as a T-score with a mean of 50 and standard deviation (SD) of 10 in the general population, which makes the scales easier to interpret. Scores above 50 indicate health status equal or above the US norm (2009), and scores below 50 indicate poorer health status than the norm [14]. As a rule, group-level scores within 0.3 SD (3 T-score points) of the mean are within the normal range. Individual scores that fall below a T-score of 45 (0.5 SD) are considered to be below the average range for the population ([14] p. 71). We used the NBS score (T-scores) for descriptive purposes and comparison with the norm. Transformed 0–100 SF-12 scores were used for correlational analyses and comparison of HRQoL between groups.

The categorical data on type and localization of symptoms reported in a 5 × 5 table were used to produce a numerical symptom score for each participant. This summary score was achieved by giving each symptom a value of 1 and each of its associated localization a value of 1, thus providing a maximum score of “5 types of symptoms x 5 possible localizations” = 25. To exemplify, if a participant reported blisters and fragile skin (= two symptoms) and localization of both these symptoms were on the hands and face (= two places), this would produce a symptom score of four.

We used non-parametric tests because of the small sample size and non-normal distribution. We used Spearman’s rho correlational analysis to examine the associations between symptoms, PCS, MCS and SF-12 subdomains, and urinary uroporphyrin concentration in the overall group and in men and women analysed separately. A correlation coefficient of + .3 was defined as medium effect and + .5 as a large effect [16, 17]. The Mann–Whitney *U*-test was used to identify differences in symptoms, urinary uroporphyrins, PCS, MCS and SF-12 subdomains between groups as defined by sex, type of PCT, initiated/ongoing treatment, smoking and alcohol consumption.

We used QualityMetric health outcomes™ Scoring Software 4.0 [18] to calculate all SF-12 scores, both T-scores and 0–100 scores. All other statistical analyses were performed using IBM SPSS Statistics Version 24. *P*-values less than 0.05 were considered significant.

## Results

### Study population

One hundred and eleven participants were given a diagnosis of PCT between December 2013 and December 2015. Sixty-eight of these completed the questionnaire and seven were excluded (> 6 months from time of drawing samples resulting in the porphyria diagnosis to answering the questionnaire; *n* = 6, missing data on treatment initiation; *n* = 1), giving in total 61 participants in the final analyses (55%). Participant characteristics are shown in Table 1. A similar number of men and women participated; their median age was 60 years (range 24–79). One-third reported smoking and about 80% reported low to moderate alcohol consumption (Table 1). Of 43 non-responders, 27 (63%) were males and 16 (37%) women. Median age was 58 years (range 23–83).

**Table 1:** Participant characteristics and biochemical markers

	*N* = 61
Age^a^	60 (24–79)
Urinary uroporphyrin^b^	452 (128–1528)
Male	28
Female	33
Familial PCT	26
Sporadic PCT	32
Smoking	21
No smoking	39
Low-to-moderate^c^ alcohol consumption	48
Moderate-to-high^d^ alcohol consumption	11
Treatment for PCT initiated	25
Treatment for PCT not initiated	36
Highest completed education	
Primary school	14
High school	27
University and college < 4 years	15
University and college > 4 years	5
Employment	
Paid work	33
Retirement	20
Disability pension	7
Other^e^	9

^a^ Median with range, in years

^b^ Median with range, in nmol/mmol creatinine

^c^ 0–10 units per week

^d^ ≥ 11 units per week

^e^ Long-term sick leave, staying at home, in education or military service, unemployed, other

### Health-related quality of life

**Fig. 1:**
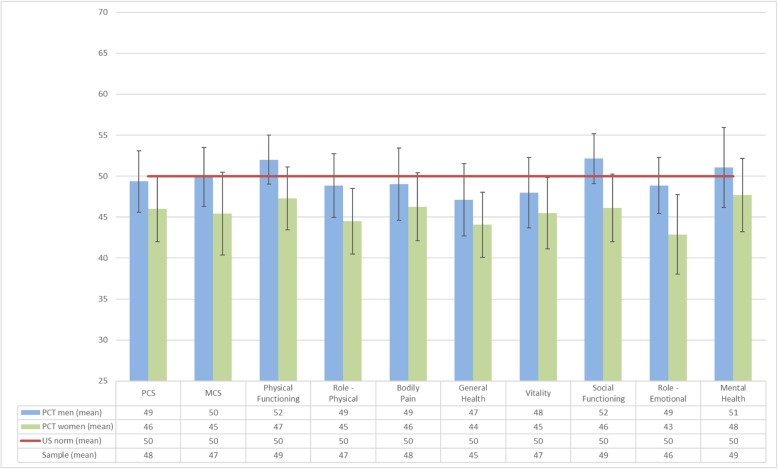
Norm-based scores (T-scores) with 95% confidence intervals for PCS, MCS and SF-12 subdomains in the total sample, and for men and women separately compared with the US 2009 norm. Group-level scores between 47 and 53 are within the normal range. The norm based t-scores have a mean of 50 and a standard deviation of 10 [14]

HRQoL scores for the overall group for both the PCS (mean 48) and MCS (mean 47) were similar to the US norm. By contrast, for scores relating to general health, the participants had lower scores (mean 45), and they reported more problems with work and other activities because of emotional problems (mean 46) than the norm (Fig. 1). Twenty-one participants (34%) had a PCS score below 45, and 24 (39%) had a MCS score below 45 (data not shown). Scores between 45 and 55 are considered to be within the normal range in individual respondents [14].

Women reported lower HRQoL compared with men on both summary scores (PCS and MCS) and six of eight health domains (Fig. 1). However, this difference was significant only for social functioning (Table 2). Participants who had started treatment (*n* = 25) before answering the questionnaire had a significantly higher PCS score than those who had not started treatment (Table 2). HRQoL scores did not differ significantly between participants with sporadic and familial PCT, smokers and non-smokers, those with low-to-moderate and moderate-to-high alcohol intake (data not shown).

**Table 2:** Mann–Whitney *U* test analysis of SF-12 0–100 scores, biochemical markers and symptom scores between women vs. men and treatment initiated vs. treatment not initiated

	*N*	Median	Quartiles25–75%	*P-*valueMann–Whitney	Cohen’s d	Mean	Min–max	SD
**SF-12** PCS								
Treatment initiated	25	87	64–96	.045*	.50	75	19–96	26
Treatment not initiated	35	71	38–90			61	6–100	30
Bodily pain								
Treatment initiated	25	100	75–100	.013*	.59	83	0–100	30
Treatment not initiated	36	75	31–100			64	0–100	34
Social functioning								
Women	33	75	38–100	.041*	.59	70	0–100	34
Men	28	100	75–100			87	25–100	23
**Biochemical markers**								
Uroporphyrins^a^								
Women	33	557	286–941	.022*	.62	625	145–1528	370
Men	28	369	215–531			426	128–1018	263
**Symptoms**								
Symptoms score^b^								
Women	33	6	3–8	.042*	.79	6	0–15	3
Men	28	3	2–6			4	1–9	2

Mann–Whitney *U* test was performed for all variables in all groups. Only results associated with a p-value < 0.05 are shown. Groups tested: familial PCT vs. sporadic PCT, low-to-moderate alcohol consumption vs. moderate-to-high alcohol consumption, smoking vs. no smoking

The following SF-12 domains were also tested but no significant differences between groups were identified: MCS, role physical, general health, vitality, role emotional, mental health

^a^Uroporphyrins: urinary excretion of uroporphyrins in nmol/mmol creatinine

^b^Symptoms: numerical score based on number and localization of symptoms (0–25)

* *p* < .05; ** *p* < .01

Analysis of the associations between self-reported symptoms and HRQoL showed a moderate negative relationship between PCT symptoms and both mental and physical aspects of HRQoL. Separate analyses for men and women showed a moderate association between symptoms, MCS and PCS in women, and strong associations between symptoms and PCS and general health in men (Table 3).

**Table 3:** Spearman’s rho correlational analysis between urinary uroporphyrin concentration, symptoms, and SF-12 0–100 scores

	Total		Women		Men	
	Uroporphyrins	Symptoms	Uroporphyrins	Symptoms	Uroporphyrins	Symptoms
Symptoms	.219		.023		.344	
PCS	−.202	−.441**	−.133	−.364*	−.284	−.614**
MCS	−.186	−.354**	−.129	−.353*	−.230	−.379
Physical functioning	−.178	−.350**	−.105	−.343	−.206	−.352
Role physical	−.258*	−.406**	−.152	−.414*	−.342	−.422*
Bodily pain	−.179	−.291*	−.147	−.251	−.266	−.392*
General health	−.133	−.372**	−.014	−.258	−.265	−.574**
Vitality	−.007	−.283*	.033	−.356*	−.055	−.230
Social functioning	−.143	−.326*	−.108	−.258	−.072	−.421*
Role emotional	−.230	−.341**	−.131	−.348*	−.306	−.322
Mental health	−.246	−.385**	−.221	−.341	−.304	−.464*

* *p* < .05; ** *p* < .01

### Symptoms, susceptibility factors and treatment

Almost all study participants reported blisters (95%) and sore/fragile skin (84%), followed by itching (59%), abnormal hair growth (36%) and increased pigmentation (25%) (Table 4). Other symptoms included red urine (74%) and non-skin symptoms (25%), such as tiredness, fatigue, warm and restless legs, poor appetite, stomach pain and constipation, which indicated that some patients attribute symptoms other than skin manifestations to PCT. The most commonly reported localizations of symptoms were arms/hands (98%), followed by face (57%) and legs/feet (48%) (Table 4).

**Table 4:** The percentage of participants who reported the different PCT symptoms and their localization

	Skin blisters	Sore/fragile skin	Increased pigmentation	Abnormal hair growth	Itching	Total
Arms/hands	95%	80%	13%	15%	46%	98%
Face	20%	31%	16%	33%	12%	57%
Legs/feet	21%	25%	3%	2%	18%	48%
Upper body incl. Neck	8%	7%	8%	5%	18%	31%
Other places	5%	2%	0%	2%	8%	12%
Total	95%	84%	25%	36%	59%	

Sequencing of the *UROD* gene detected a disease-associated variant in 26 participants, who were thus classified as having familial PCT. In 32 participants, no disease-associated *UROD* variant was detected (sporadic PCT) (Table 1), and in three participants, *UROD* sequencing was not performed. Of the 61 participants, 25 reported having started treatment (13 women and 12 men). Treatment was initiated a maximum of four calendar months before completing the questionnaire. Eight participants received treatment for less than a month. Four, eight and five participants had received treatment for two, three and four calendar months, respectively, before completing the questionnaire (data not shown). In the treatment-initiated group, 18 participants received venesection (8 women, 10 men), and seven received venesection in combination with medication (5 women, 2 men).

Women reported significantly more symptoms than men (Table 2). Compared to men, women also reported a significantly shorter time-gap from when the symptoms started till they sought medical attention (median: women 1 month, men 3 months, Mann-Whitney test *p* < 0.01). Women also reported less time from symptoms to time of first sampling (median: women 4.5 months, men 6 months, Mann-Whitney test *p* = 0.21). There were no differences between men and women in smoking, alcohol consumption and *UROD* status (results not shown). High alcohol consumption was the most frequently self-reported precipitating factor (25%), followed by the use of oestrogen (12%) and iron supplements (10%), and the presence of haemochromatosis and hepatitis C infection (6%, respectively).

### Disease activity and biochemical markers

Participants had a median urinary excretion of uroporphyrins of 452 nmol/mmol creatinine at the time of first sample resulting in a porphyria diagnosis (range 128–1528 nmol/mmol creatinine) (Table 1). Men had significantly lower levels of urinary uroporphyrin (median 369 nmol/mmol creatinine) compared with women (median 557 nmol/mmol creatinine, *p* = .022) (Table 2). Urinary uroporphyrin concentration did not differ significantly between those with familial vs. sporadic PCT, smokers vs. non-smokers, and those with a low-to-moderate and moderate-to-high alcohol intake (results not shown). No association between urinary uroporphyrin excretion and the symptom score was identified (Table 3).

## Discussion

One of the aims of our study was to describe HRQoL in people with PCT, and to the best of our knowledge this is the largest sample of PCT patients surveyed about their HRQoL. We used a standardized measure of HRQoL, and compared scores with the US norms. The PCS and MCS did not indicate a lower HRQoL in the overall PCT group. The appropriateness of using US-derived scoring algorithms has been demonstrated in large general population samples from nine countries, including Norway ([14] p. 42). The SF-12 is a generic instrument that gives us the opportunity to compare HRQoL in different disease groups. To our knowledge, however, only few studies have published scores from SF-12 in other relevant skin diseases [19–23]. It is a general problem that a clear presentation of the scoring algorithms are missing, resulting in erroneous conclusions because of the mixing of T-scores and 0–100 scores ([14] p. 164). This makes comparisons with our findings difficult. However, a cross-sectional study in patients suffering from chronic plaque psoriasis, reported an identical mean T-score of MCS, but a lower PCS T-score, compared to our sample [19]. This indicates that the PCT patients’ mental aspect of HRQoL are in line with the psoriasis study.

Analysis of the eight health domains showed that PCT patients had lower scores for general health (mean 45) and emotional role (mean 46) (Fig. 1) than the US norm. It is possible that underlying health issues (of unknown cause in this sample) act as susceptibility factors for PCT or are associated with PCT. Comorbid health conditions were, however, not assessed, and all the reported symptoms may not be associated with PCT.

In the overall group, we observed a moderate negative correlation between reported symptoms of PCT and physical and mental summary scores, as well as the six single domains. In men, the associations between symptoms and both PCS (*r* = **−**.614, *p* < 0.01), and general health (*r* = **−**.574, *p* < 0.01) was strong (Table 3). Previous research has not supported an association between PCT symptoms and porphyria-related distress [11], and another study concluded that PCT seems to have little impact on QoL [12]. However, the latter study included only 12 participants with PCT, most of whom were likely in clinical remission, and those results may have limited relevance for symptomatic patients.

Previous research on photosensitive disorders has shown that, despite their diverse aetiologies, these conditions can have a high psychological impact [24], including increased anxiety, depression, psychological distress [25, 26], reduced psychological well-being, and impact on lifestyle [27]. In addition, blistering diseases are associated with lower QoL, shame and the impression of poor hygiene [28]. PCT is a treatable, non-fatal condition, but the patients should avoid sunlight when symptomatic, and susceptibility factors both in the active disease phase and when in remission. Developers of guidelines for monitoring PCT should consider the subjective aspects of the condition in addition to the therapeutic outcomes. Although we cannot draw conclusions of causality in the present study, our results show that symptomatic PCT is associated with reduced HRQoL. This suggests that appropriate follow-up, such as annual assessments, to prevent and detect relapses is important to improve patient outcomes.

Of the 61 participants, 25 reported having started treatment before answering the questionnaire. Clinical and biochemical remission may take up to 13 months [7, 8]. Although 12 of the 25 reported having started treatment less than two months ago, and none had received treatment for more than four calendar months, we did observe a significant difference in HRQoL between those having started and those who had not started treatment (Table 2). These differences were related to bodily pain, and suggests that treatment may induce, at least from a subjective point of view, rapid improvement, as has been reported previously [10].

Urinary porphyrin concentrations are used to diagnose and monitor disease activity in people with PCT. Most of the porphyrins in the urine of PCT patients are uroporphyrin, and we, therefore, used uroporphyrin concentration as a biochemical indicator of disease activity. PCT patients can show a keen interest in laboratory analyses and test results [10], and it is likely that they expected their urinary concentrations to reflect the severity of symptoms. However, we found no associations between urinary uroporphyrin concentration and self-reported occurrence and localization of PCT symptoms. However, the localization of symptoms does not necessarily reflect the severity of the skin symptoms and additionally, it is likely that there may be individual thresholds for urinary uroporphyrins to trigger photosensitization, and the distribution of skin symptoms will also depend on the degree of light exposure.

We observed that men had significantly lower urinary uroporphyrin at the time of sampling compared with women (Table 2), in spite of the fact that women sought medical attention for their PCT symptoms quicker than men. We found no differences in urinary excretion between participants with familial and sporadic PCT or between any other groups (smoking, alcohol). Our observed sex difference in urinary uroporphyrin concentrations may be an incidental finding or may be related to hormonal factors, as UROD activity is influenced by oestrogen [29]. Eight women reported oestrogens as a precipitating factor.

Women reported more PCT symptoms and lower QoL than men. Women in general are known to report lower QoL [30] and more subjective health complaints [31, 32]. Analysis of the associations between self-reported symptoms and HRQoL showed a strong association between symptoms and PCS in men, while this association was only moderate in women (Table 3) The association between mental aspects and HRQoL was not present in men, and only moderately in women, which is in line with the results of a previous study on illness perception in PCT, where sex was not associated with porphyria related psychological distress [11]. Based on the data from the present study, we can therefore not draw any conclusions in regards to gender differences in the subjective experiences of PCT.

Itching was the third most reported symptom, and more than half of the participants (59%) reported this (Table 4). Itching is associated with reduced HRQoL [33–36], and although previously reported [10, 37], it is rarely included in descriptions of PCT, and physicians need to address this in this patient group. This is particularly important since itching may damage already frail skin, which can increase risk of infections and delay healing.

### Limitations

We found that symptoms of PCT were associated with reduced HRQoL (Table 3). However, the cross-sectional design precludes us from drawing conclusions about causality.

Another limitation is that we did not measure the severity of symptoms on a scale, and therefore all symptoms are scored as equal in severity, which is not necessarily the case. Consequently, we do not know whether the scoring method in this study accurately reflects the patients’ perception of the severity of symptoms, and the Norwegian Porphyria Registry will evaluate this mode of measuring symptoms in the future. However, our data allow us to conclude that occurrence and localization of symptoms were associated with reduced HRQoL in these patients.

A time gap of up to six months between the time of sampling and answering the questionnaire represents a limitation of the study. However, participants were unlikely to learn about their PCT diagnosis before three to 10 weeks after the sampling date, and the median number of days from sampling to completing the questionnaire was 70 (range 21–167 days). In addition, the participants were asked about which symptoms they *had* experienced in connection to PCT and the SF-12 has a standard four-week recall period. Still, for some participants, recall bias might have affected their responses.

The number of non-responders is relatively high and the present study had a response rate of 55%, which is a potential problem in regard to representability. This is, however, a common problem and average response rates have been estimated to approximately 53% [38]. We did not perform any further analyses of the non-respondents because according to our Ethical Committee, such data extraction and analyses cannot be performed without consent.

We used a generic measure of HRQoL (SF-12), but a dermatology-specific instrument may have been more appropriate. However, the SF has been recommended as a generic instrument that is well suited to dermatology, preferably in combination with the Skindex-29 [39].

We performed multiple tests of many groups, increasing the possibility of a chance finding. Unfortunately, our sample size was too small to perform any adjustments for multiplicity. We did, however, conduct non-parametric tests to better model the small sample sizes we had per group, which have lower study power than standard tests.

## Conclusions

We observed a moderate negative correlation between reported symptoms and HRQoL. PCT is a non-fatal and treatable condition, but it may require lifelong follow-up and avoidance of susceptibility factors to avoid relapses. Our results indicate that annual follow-up could be important for avoiding relapses that might negatively influence the patients’ HRQoL. Itching has traditionally been given little focus in PCT, but it was reported by 59% of participants and was the third most common symptom of PCT. To avoid the increased risk of infections and delay in healing, symptoms of itching and treatment options should be addressed in patient consultations.

Our results underline the importance of starting treatment as soon as possible after diagnosis. PCT is a rare disease, and many physicians will have little or no experience with the best ways to treat and follow up such patients. This highlights the need for the development of best-practice guidelines to facilitate good patient care.

## Data Availability

Please contact author for data request.

## Abbreviations

PCT: Porphyria cutanea tarda
UROD: Uroporphyrinogen decarboxylase
PRO: Patient reported outcome
QoL: Quality of life
HRQoL: Health-related quality of life
NAPOS: The Norwegian Porphyria Centre
SF-12: Short Form-12 Health Survey version 2
PCS: Physical Component Summary
MCS: Mental Component Summary
NBS: Norm-based scoring
